# Supplementary material for: Carbon nitride caught in the act of artificial photosynthesis
Source: Nat Commun. 2025 Jan 3;16:374. doi: 10.1038/s41467-024-55518-x (PMC11698875; doi:10.1038/s41467-024-55518-x)
Supplement: Supplementary file 1 — Supplementary Information [file 41467_2024_55518_MOESM1_ESM.pdf]

Supplementary Information

Carbon Nitride Caught in the Act of Artificial Photosynthesis

Daniel Cruz[1]†, Sonia Zoltowska[2]†, Oleksandr Savateev[2], Markus Antonietti[2], and Paolo Giusto[2*]

Corresponding author: Paolo.Giusto@mpikg.mpg.de

**Supplementary information**	**Page**
Preliminary tests	3
Figure S1	4
Figure S2	5
Figure S3	6
Figure S4	7
Figure S5	8
Figure S6	9
Figure S7	10
Figure S8	11
Figure S9	12
Figure S10	13
Figure S11	14
Figure S12	15
Figure S13	16
Supplementary note 1	17
Supplementary note 2	21
Supplementary note 3	25
Supplementary note 4	27
Supplementary note 5	28
Supplementary References	29

Preliminary tests

Prior to test the carbon nitride thin films in presence of water and light we characterize the as-prepared sample in its bare state using the same experimental conditions as for all the other spectroscopic experiments. The XPS characterization of the bare sample reveals the typical features of carbon nitride thin films[1-5] (Figure S2 A, B), with a carbon-to-nitrogen ratio of 0.74, very close to the ideal 0.75. NEXAFS spectra of the C and N-edges (Figure S2 C, D) are in good agreement with previous theoretical and experimental reports on carbon nitride materials.[5,6] It is worth noticing the presence of adventitious carbon, i.e. carbon material unintentionally adsorbed on the sample surface,[2] appearing as a C-C peak in the XPS (285.3 eV) and NEXAFS (285.0 eV).[1,2,5,6]. VB-XPS reveal HOMO values to be consistent with previously reported values (+1.59 eV) (Figure S2 E)[1]. Based on these data, we propose the structure of carbon nitride thin films based on heptazine as a main repeating unit with $-NH_x$ terminal groups, such as -NH- and $-NH_2$ (Figure S2 F).

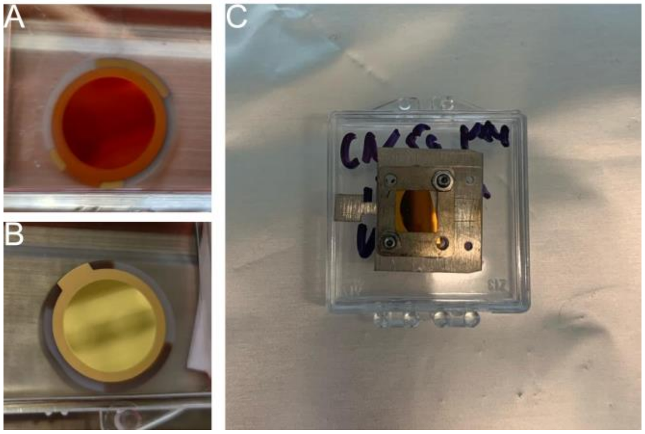

Figure S1. Pictures of the samples used in this study. (A) carbon nitride thin film obtained via chemical vapor deposition; (B) gold reference substrate; (C) carbon nitride thin film assembled in the holder used for the following characterization.

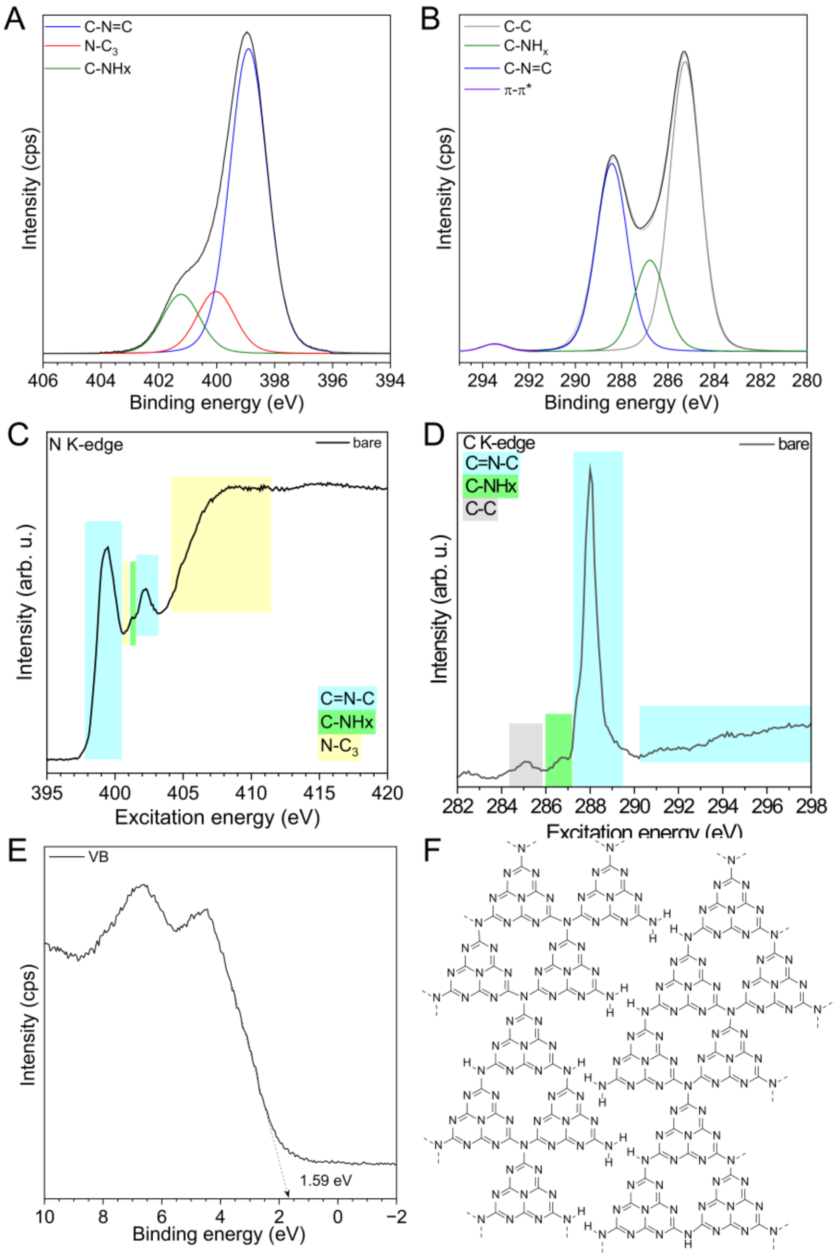

Figure S2. Spectroscopic features of the carbon nitride thin film. (A) XPS, N1s spectra of carbon nitride thin film bare. (B) XPS, C1s spectra of carbon nitride thin film. (C) NEXAFS, N K-edge of the carbon nitride thin film bare. (D) NEXAFS, C K-edge of the carbon nitride thin film bare. (E) VB-XPS of the carbon nitride thin film bear with relative HOMO value. (F) Schematic representation of the bare carbon nitride structure.

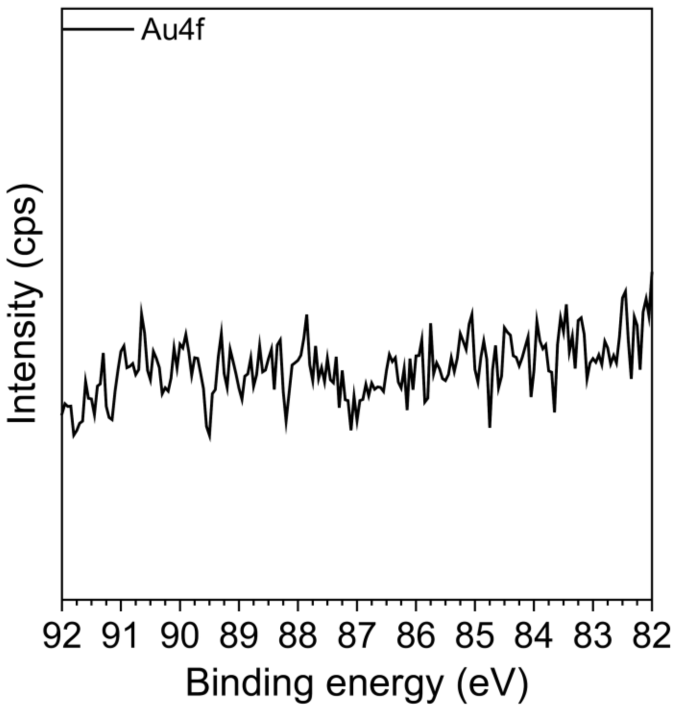

Figure S3. XPS of carbon nitride thin film at the Au4f binding energy range. Au4f XPS spectrum of bare carbon nitride thin film (no background subtraction applied).

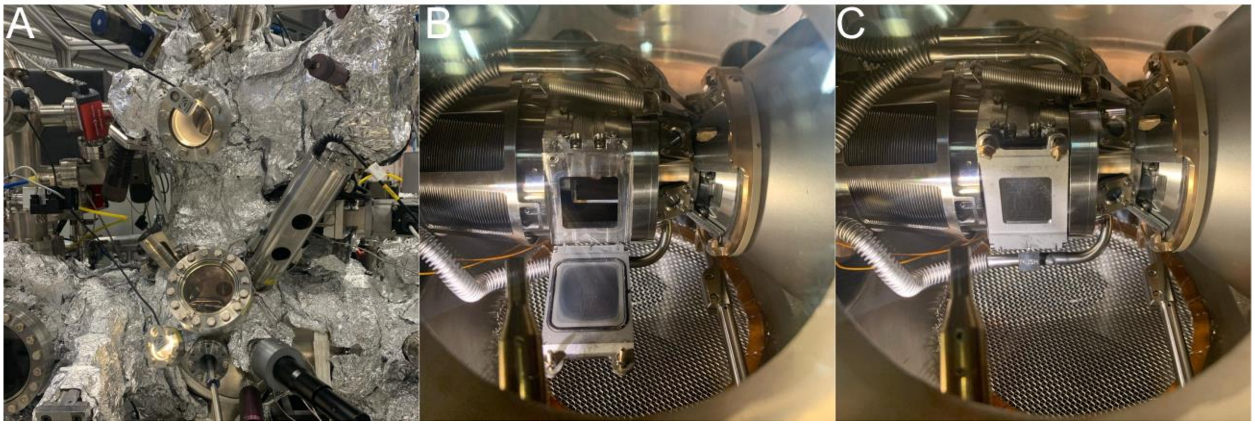

Figure S4. Pictures of the synchrotron device and cell used for the characterizations. (A) external view (B) unit for the allocation of the sample open and (C) closed.

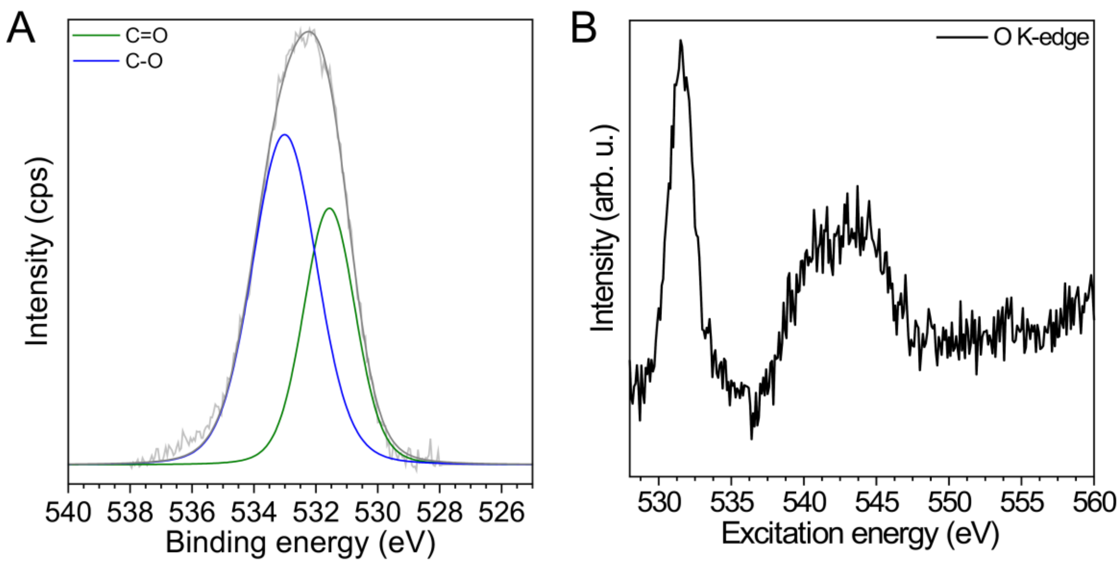

Figure S5. Characterization of oxygen bonding structures via XPS and NEXAFS. (A) O1s XPS spectra of the bare carbon nitride thin film; (B) O K-edge of the carbon nitride thin film bare. These features are usually attributed to the presence of adventitious species on the surface of the sample.[7-9]

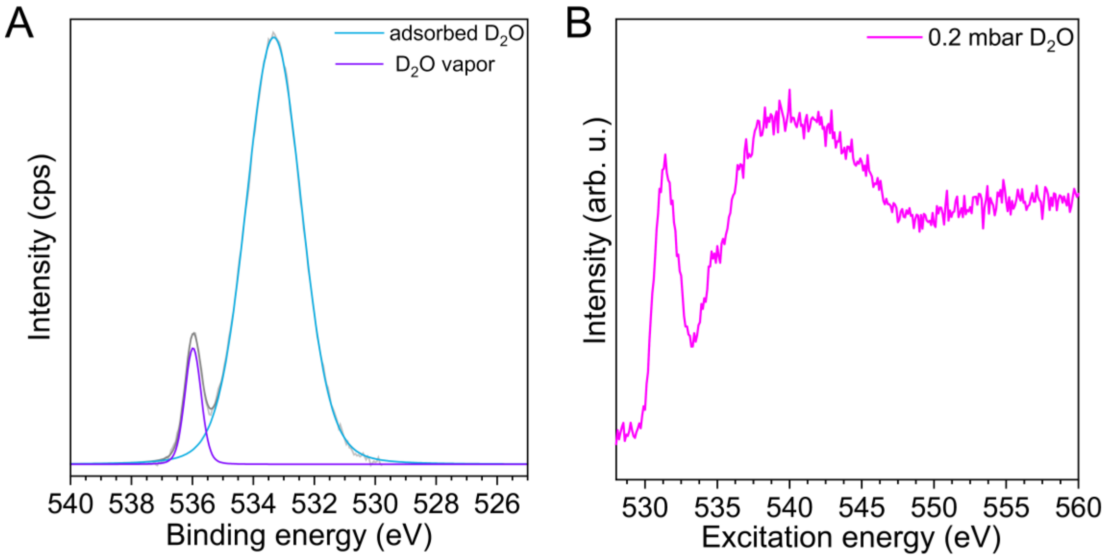

Figure S6. In-situ characterization of oxygen bonding structures via XPS and NEXAFS during water adsorption. (A) O1s XPS spectra of the bare carbon nitride thin film exposed to 0.2 mbar D_2O; (B) O K-edge of the carbon nitride thin film exposed to 0.2 mbar D_2O.

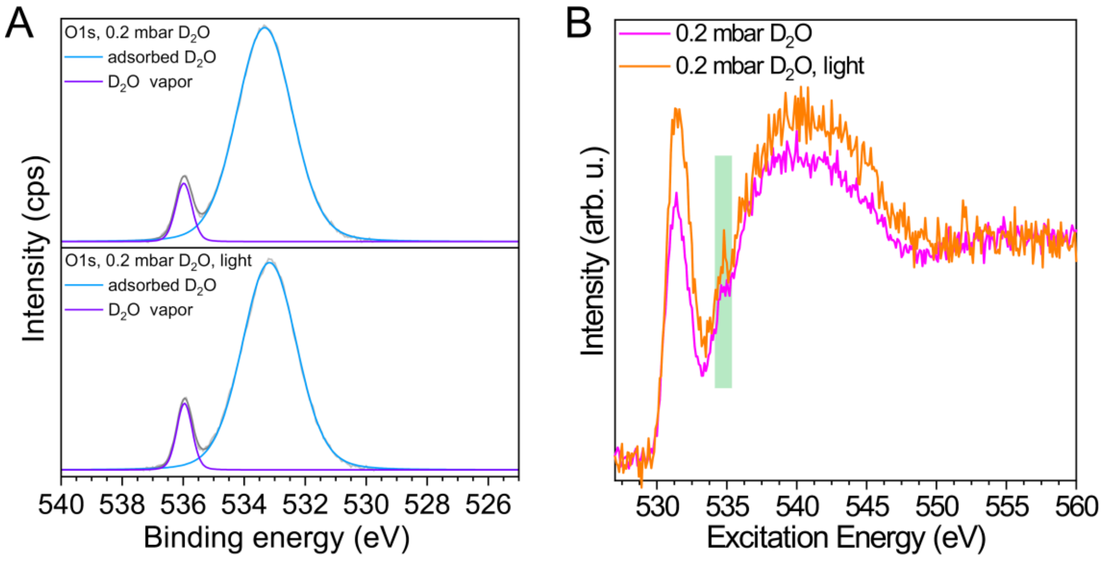

Figure S7. In-situ characterization of oxygen bonding structures via XPS and NEXAFS during water adsorption and light illumination. (A) O1s XPS spectra of carbon nitride thin film exposed to 0.2 mbar D_2O (top panel) and carbon nitride thin film exposed to 0.2 mbar D_2O and light irradiation (bottom panel). (B) O K-edge of the carbon nitride thin film exposed to 0.2 mbar D_2O (magenta line) and light irradiation (orange line). The arising sharp peak, upon light illumination, at 534.8 eV (green box) is attributed to O1s $\rightarrow \sigma^*$ ($4a_1$).[10]

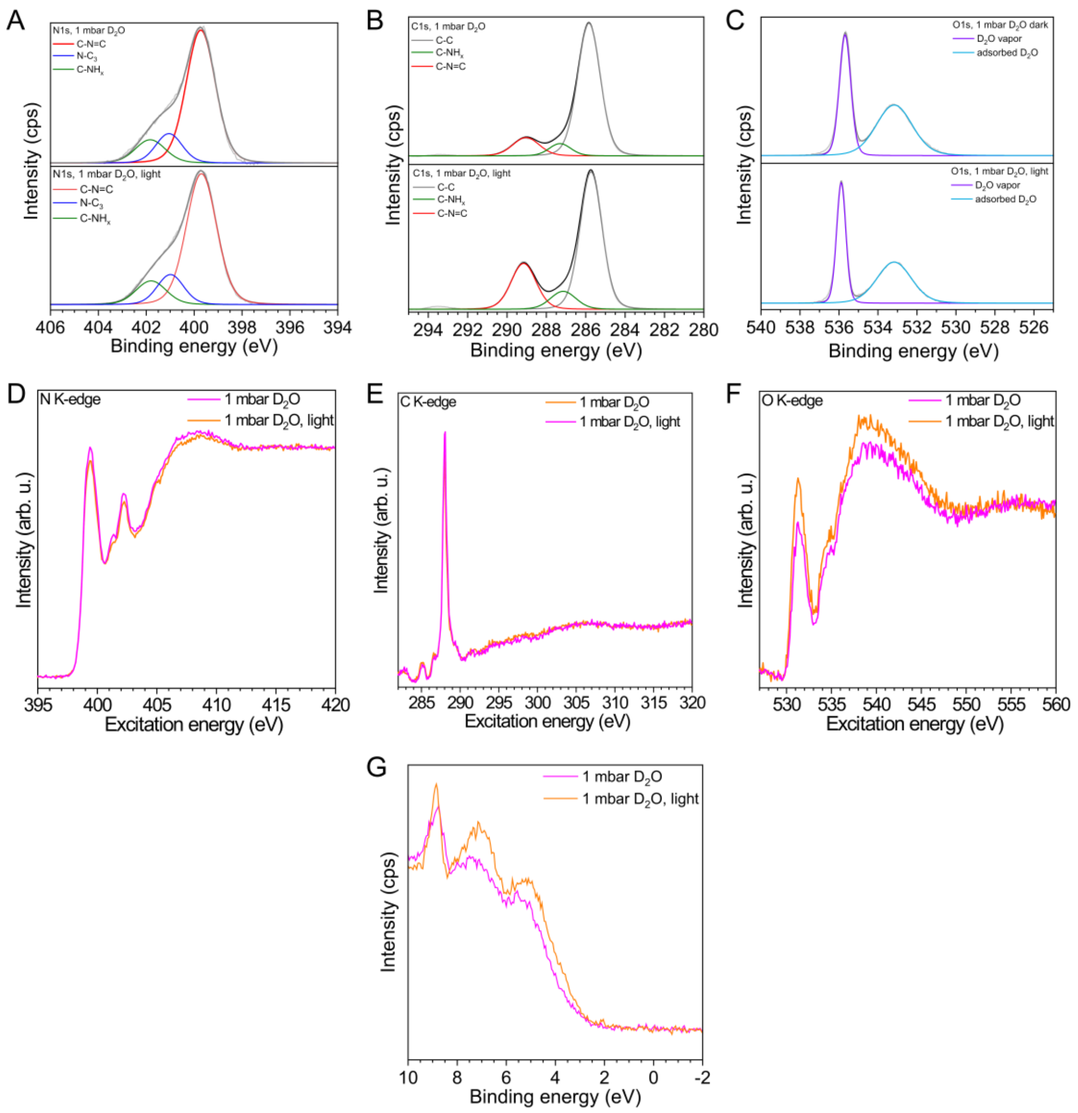

Figure S8. In-situ adsorption of heavy water on the carbon nitride thin film surface. (A) XPS, N1s spectra of carbon nitride thin film exposed to 1 mbar D_2O (top panel) and carbon nitride thin film exposed to 1 mbar D_2O and light irradiation (bottom panel). (B) XPS, C1s spectra of carbon nitride thin film exposed to 1 mbar D_2O (top panel) and carbon nitride thin film exposed to 1 mbar D_2O and light irradiation (bottom panel). (C) XPS, O1s spectra of carbon nitride thin film exposed to 1 mbar D_2O (top panel) and carbon nitride thin film exposed to 1 mbar D_2O and light irradiation (bottom panel). (D) NEXAFS, N-K edge of the carbon nitride thin film exposed to 1 mbar D_2O (magenta line) and carbon nitride thin film exposed to 1 mbar D_2O and light irradiation (orange line). (E) NEXAFS, C-K edge of the carbon nitride thin film exposed to 1 mbar D_2O (magenta line) and carbon nitride thin film exposed to 1 mbar D_2O and light irradiation (orange line). (F) NEXAFS, O-K edge of the carbon nitride thin film exposed to 1 mbar D_2O (magenta line) and carbon nitride thin film exposed to 1 mbar D_2O and light irradiation (orange line). (G) VB-XPS of the carbon nitride thin film exposed to 1 mbar D_2O (magenta line) and carbon nitride thin film exposed to 1 mbar D_2O and light irradiation (orange line).

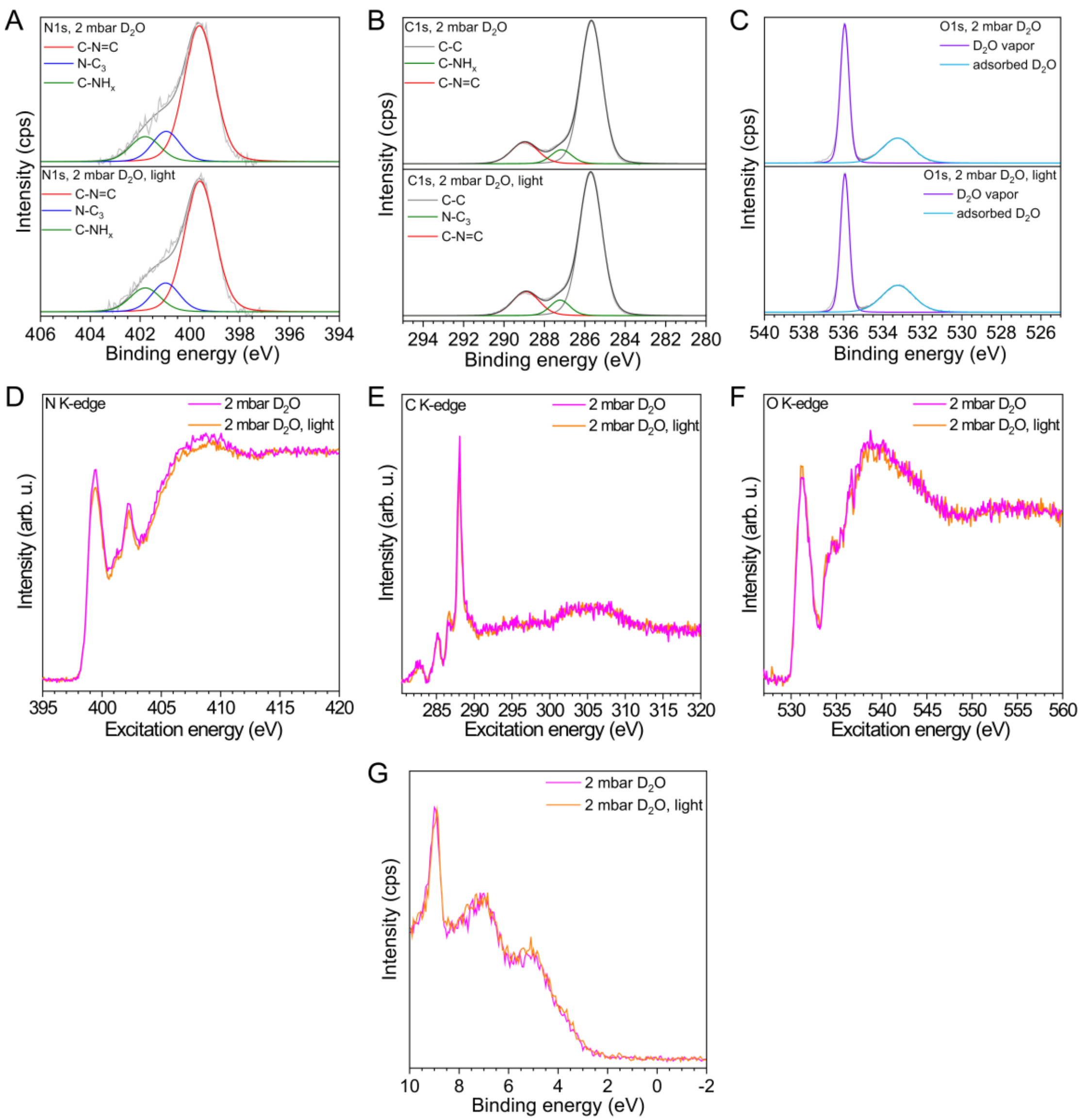

Figure S9. In-situ adsorption of heavy water on the carbon nitride thin film surface. (A) XPS, N1s spectra of carbon nitride thin film exposed to 2 mbar D_2O (top panel) and carbon nitride thin film exposed to 2 mbar D_2O and light irradiation (bottom panel). (B) XPS, C1s spectra of carbon nitride thin film exposed to 2 mbar D_2O (top panel) and carbon nitride thin film exposed to 2 mbar D_2O and light irradiation (bottom panel). (C) XPS, O1s spectra of carbon nitride thin film exposed to 2 mbar D_2O (top panel) and carbon nitride thin film exposed to 2 mbar D_2O and light irradiation (bottom panel). (D) NEXAFS, N-K edge of the carbon nitride thin film exposed to 2 mbar D_2O (magenta line) and carbon nitride thin film exposed to 2 mbar D_2O and light irradiation (orange line). (E) NEXAFS, C-K edge of the carbon nitride thin film exposed to 2 mbar D_2O (magenta line) and carbon nitride thin film exposed to 2 mbar D_2O and light irradiation (orange line). (F) NEXAFS, O-K edge of the carbon nitride thin film exposed to 2 mbar D_2O (magenta line) and carbon nitride thin film exposed to 2 mbar D_2O and light irradiation (orange line). (G) VB-XPS of the carbon nitride thin film exposed to 2 mbar D_2O (magenta line) and carbon nitride thin film exposed to 2 mbar D_2O and light irradiation (orange line).

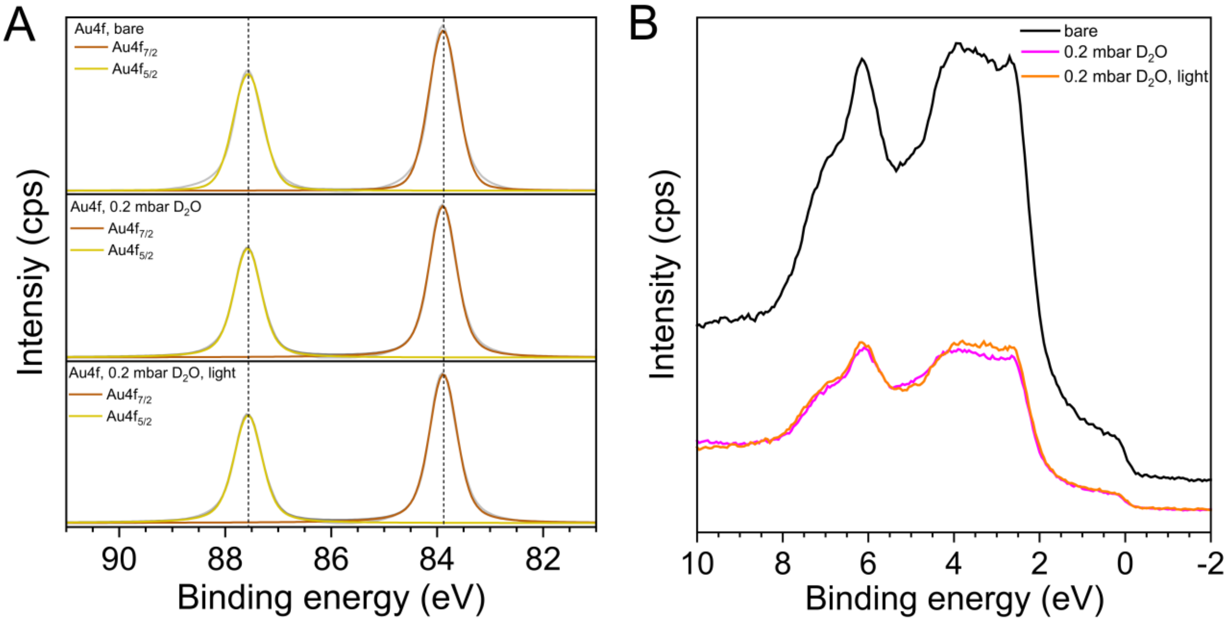

Figure S10. In-situ characterization of gold bonding structures. (A) Au4f XPS spectrum of Au substrate in vacuum (top panel), exposed to 0.2 mbar D_2O (middle panel), and exposed to 0.22 mbar D_2O and light irradiation (lower panel). (B) VB-XPS of the Au substrate bare (black line), exposed to 0.2 mbar D_2O (magenta line) and exposed to 0.2 mbar D_2O and light irradiation (orange line).

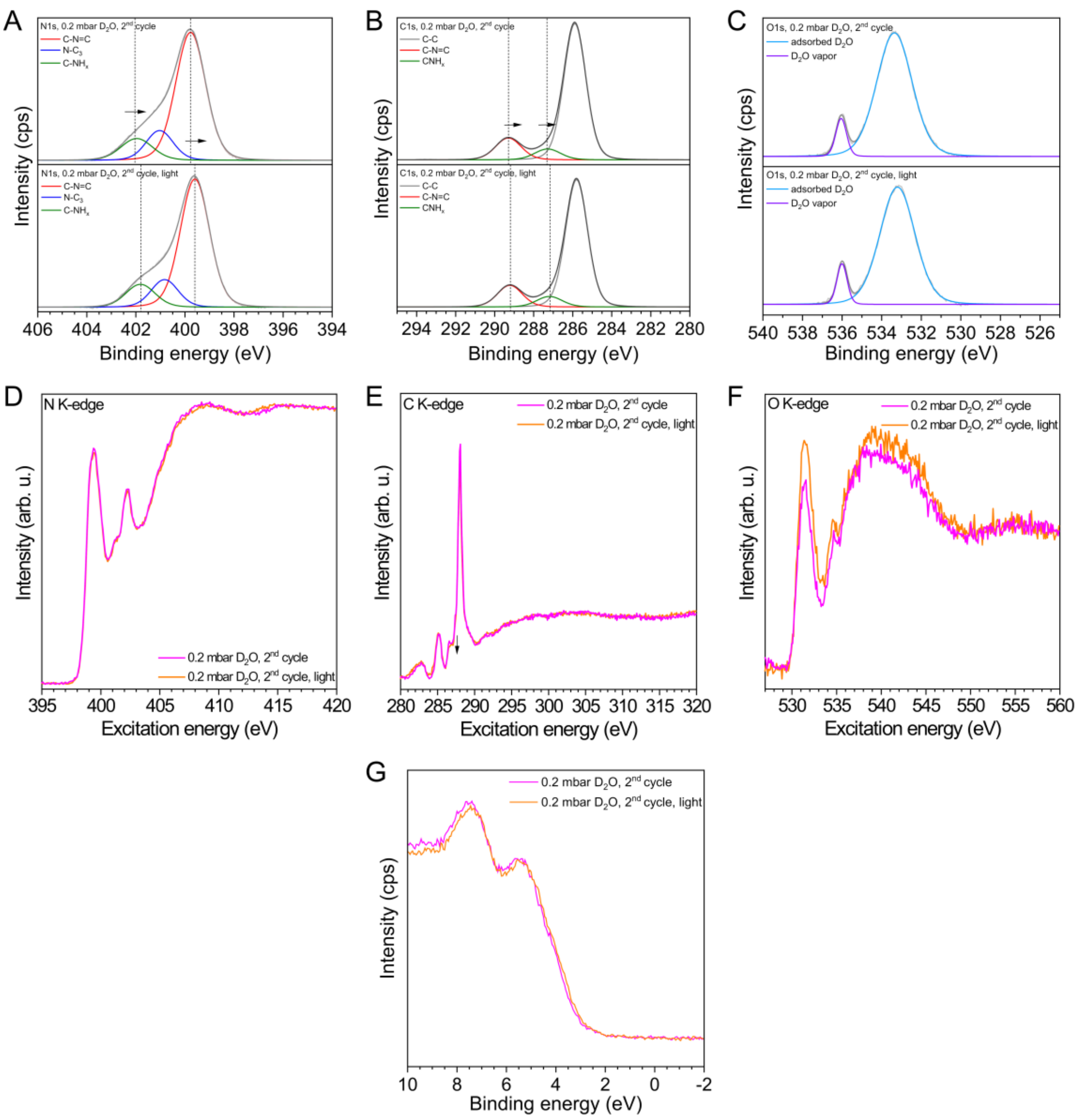

Figure S11. In-situ spectroscopic features of the second illumination cycle. (A) XPS, N1s spectra of carbon nitride thin film exposed to 0.2 mbar D_2O (top panel) and carbon nitride thin film exposed to 0.2 mbar D_2O and light irradiation (bottom panel). (B) XPS, C1s spectra of carbon nitride thin film exposed to 0.2 mbar D_2O (top panel) and carbon nitride thin film exposed to 0.2 mbar D_2O and light irradiation (bottom panel). (C) XPS, O1s spectra of carbon nitride thin film exposed to 0.2 mbar D_2O (top panel) and carbon nitride thin film exposed to 0.2 mbar D_2O and light irradiation (bottom panel). (D) NEXAFS, N-K edge of the carbon nitride thin film exposed to 0.2 mbar D_2O (magenta line) and carbon nitride thin film exposed to 0.2 mbar D_2O and light irradiation (orange line). (E) NEXAFS, C-K edge of the carbon nitride thin film exposed to 2 mbar D_2O (magenta line) and carbon nitride thin film exposed to 0.2 mbar D_2O and light irradiation (orange line). (F) NEXAFS, O-K edge of the carbon nitride thin film exposed to 0.2 mbar D_2O (magenta line) and carbon nitride thin film exposed to 0.2 mbar D_2O and light irradiation (orange line). (G) VB-XPS of the carbon nitride thin film exposed to 0.2 mbar D_2O (magenta line) and carbon nitride thin film exposed to 0.2 mbar D_2O and light irradiation (orange line).

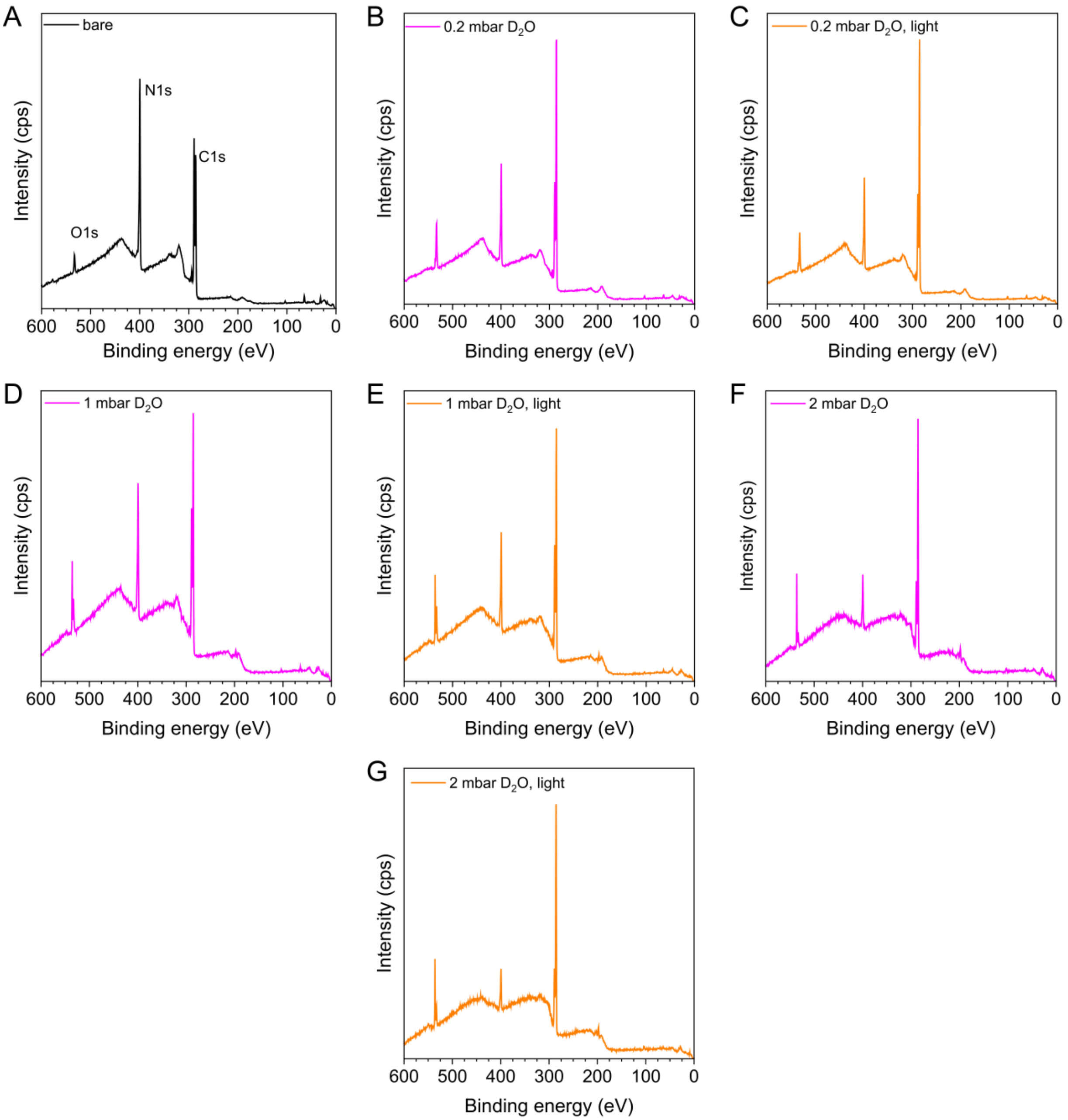

Figure S12. XPS overview of the carbon nitride film. (A) bare, (B) exposed to 0.2 mbar D_2O, and (C) exposed to 0.2 mbar D_2O and light irradiation; (D) exposed to 1 mbar D_2O, and (E) exposed to 1 mbar D_2O and light irradiation; (F) exposed to 2 mbar D_2O, and (G) exposed to 2 mbar D_2O and light irradiation

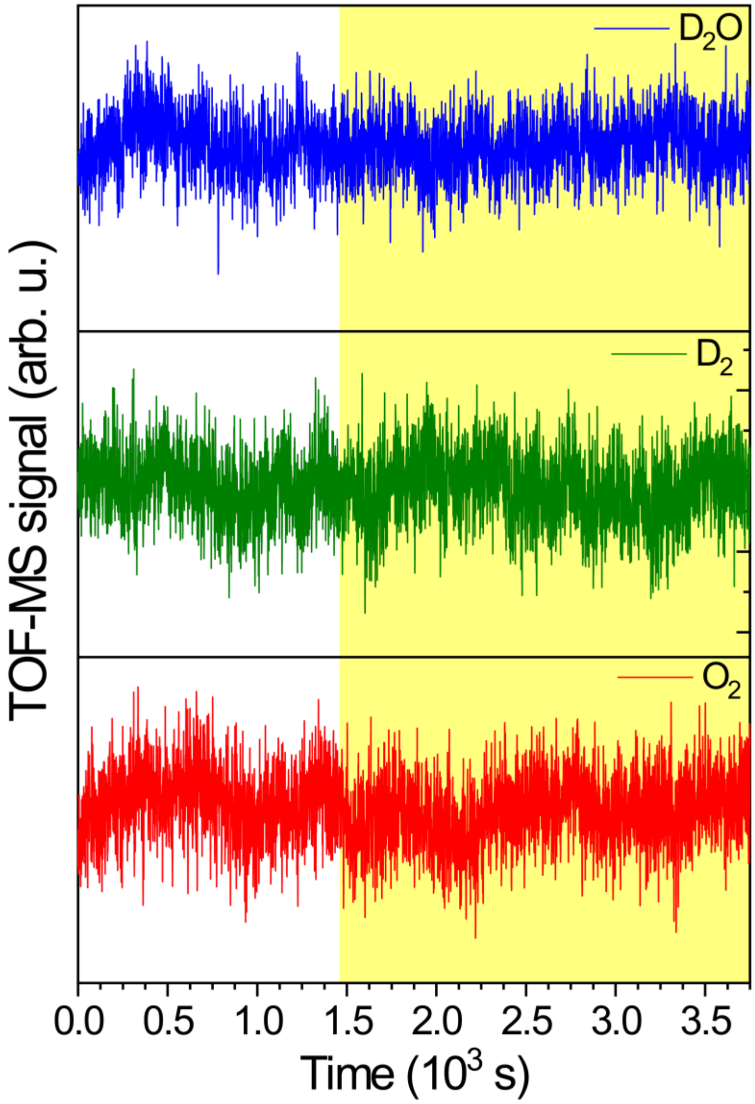

Figure S13. TOF-MS signals collected during photocatalytic study. Signal of heavy water (upper panel), D_2 (middle panel), and O_2 (lower panel); white areas indicate the absence of illumination; yellow areas indicate the time the bare gold substrate was subjected to illumination by means of the solar simulator.

Supplementary note 1:

Effect of D_2O pressure and water desorption study

It was previously shown by Wu et al.[11], that the adsorption of water at the carbon nitride occurs via the formation of hydrogen bonds and, at low coverage, is thermodynamically favored with respect to the formation water clusters. At lower coverage, occurring at lower D_2O vapor pressures, the water preferentially adsorbs at the carbon nitride surface with the heptazine's N acting as hydrogen bond donor and the negatively polarized O of the heavy water back-binds with the positively polarized heptazine's C.[12] At higher coverage, the water forms clusters on the surface of the carbon nitride, stabilized by hydrogen bonds between the water molecules.[11] To investigate the role of the adsorption of heavy water molecules on the carbon nitride electronic properties we merged the XPS and VB-XPS data stacked as a function of pressure to enable a more direct comparison between the different conditions. As shown in Figure S14A and B, a major shift of the C1s and N1s features towards higher binding energies is recorded from the bare sample the lowest heavy water vapor pressure (0.2 mbar). Notably, the elemental peaks and the deconvoluted features do not significantly shift at higher heavy water pressures, except for the C1s C-N=C feature. Here, the peak maxima at higher vapor pressures (1 and 2 mbar, 3rd and 4th panels, respectively, Figure S14A) are shifted to lower binding energies of about 0.3 eV with respect to the 0.2 mbar D2O pressure. We attribute this effect to the formation of a multilayer/cluster adsorption of water occurring when all the adsorption sites on the carbon nitride are occupied. As a result, the donation of electron density from the heavy water's O to the C of the heptazine units is reduced causing a shift to lower binding energies of the C-N=C feature in the C1s, pointing to stronger interactions for the heavy water intermolecular (O---D) than the one between heavy water and carbon nitride (C---O). While the multilayer adsorption affects the C1s spectrum at 1 and 2 mbar pressure with respect to the 0.2 mbar one, this has only a minor effect on the N1s, which is attributed to the stronger interactions in the hydrogen bond formation between the heptazine's N and the heavy water's D (N---D) with respect to heavy water intermolecular ones (O---D). This is further supported by the VB-XPS results, where at 0.2 heavy water vapor pressure the resulting VB value is shifted from 1.59 eV to 2.63 eV. The increase of vapor pressure causes an increase of this value up to 2.80 eV. This is consistent with our interpretation, since the formation of water clusters (or multilayer adsorption) reduces the back-binding effect between the heavy water's O and the heptazine's C, further reducing the electron density at the carbon nitride surface.

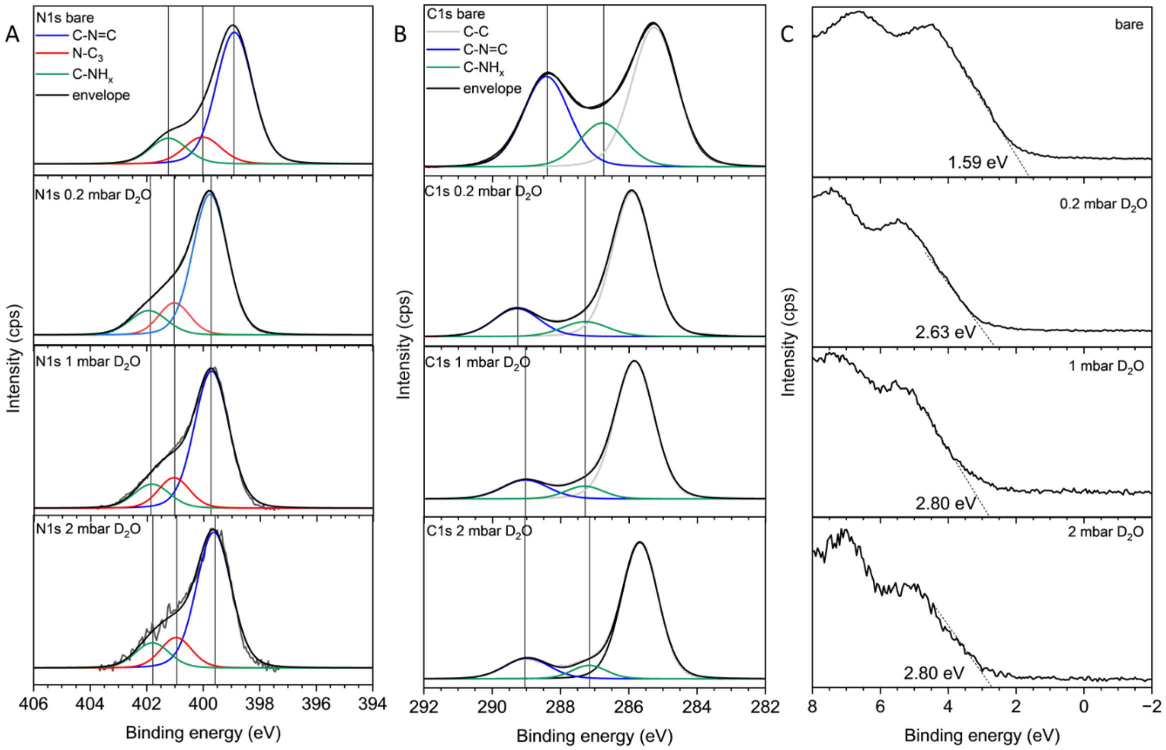

Figure S14. In-situ water adsorption study. (A) N1s, (B) C1s, (C) VB-XPS spectra of carbon nitride thin films at increasing D_2O vapor pressure. From top to bottom: bare carbon nitride film (top panel), 0.2 mbar D_2O (2nd panel), 1 mbar D_2O (3rd panel), 2mbar D_2O (4th panel).

As depicted in Figure 2 upon water adsorption, the formation of hydrogen bonds at the carbon nitride surface is primarily driven by interactions between nitrogen atoms (acting as electron donors) and water molecules. As a result, the electron density around nitrogen is reduced, causing the N1s and C1s features to shift towards higher binding energies. The water desorption step has been performed by stopping the heavy water flow and evacuating the measurement chamber in vacuum for 45 minutes before collecting the XPS spectra (Figure S15 A-C). After the chamber evacuation and water desorption, we record a negligible shift in the N1s spectra (<0.1 eV) (Figure S15 A). However, in the C1s spectra (Figure S15 B) we record a significant shift towards higher binding energies of <0.3 eV. This is attributed to the removal of more loosely bound water from the surface of the carbon nitride where only the strongest bound water molecules are retained, further depleting the electron density at the carbon. The resulting VB is also reduced and attributed to the formation of the most stable heavy water-carbon nitride system, with a VB value recorded of 2.8 eV (Figure S15 C). The results are consistent with the investigations of Wu et al.[11] revealing changes in the electronic properties of water, with the experimental results showing the same trend and consistent change in the electronic properties. Indeed, in their work, Wu et al.[11] found that the adsorption of water induces a structural (and irreversible upon desorption) change in the carbon nitride from a planar to a buckled structure, resulting in a material with a higher oxidation potential.

In their calculation, the adsorption of water leads to a higher VB value, up to 2.5 eV for a single water molecule adsorbed per unit cell.

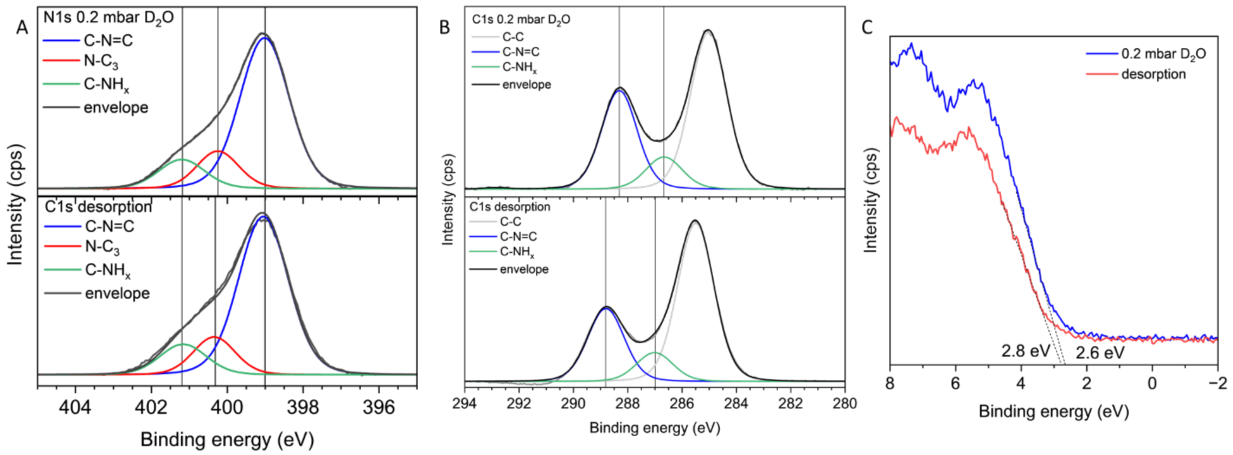

Figure S15. Water desorption study. XPS spectra of (A) N1s and (B) C1s and (C) VB-XPS of carbon nitride under flow of 0.2 mbar D_2O (middle panel) and after desorption (bottom panel).

Furthermore, XPS at increasing kinetic energy (150 keV, 400 keV and 800 keV, corresponding to 0.51 nm, 0.93 nm, and 1.5 nm penetration) spectra were collected to evaluate the effect of the water adsorption at different penetration depths on the carbon nitride thin film exposed to 0.2 mbar of heavy water. In Figure S16 A, we record a shift towards lower binding energies of about 0.3 eV. This points to a stronger effect of water adsorption on the electronic properties of the carbon nitride thin film surface rather than on the subsurface. This is further confirmed by the shift recorded at the C1s features (Figure S16 B) which shift towards lower binding energies of about 0.2 eV with respect to the lowest kinetic energy applied. Furthermore, at increasing kinetic energies, we can clearly see an increase in the areal ratio between the C-N=C features and the C-C features (attributed to the adventitious carbon) confirming that the C-C component is to be attributed to surface contamination of carbon species adsorbed on the surface. Eventually, we confirmed that the electronic properties, in terms of valence band, are significantly affected on the surface by adsorption of water and the effect is less pronounced at higher depths (Figure S16 C). Indeed, upon water adsorption the carbon nitride thin film surface valence band shifts towards higher values (2.6 eV) while at lower penetration depths it retains consistent values with the bare sample (1.6 eV) and supporting the dominant effect of water at the surface level.

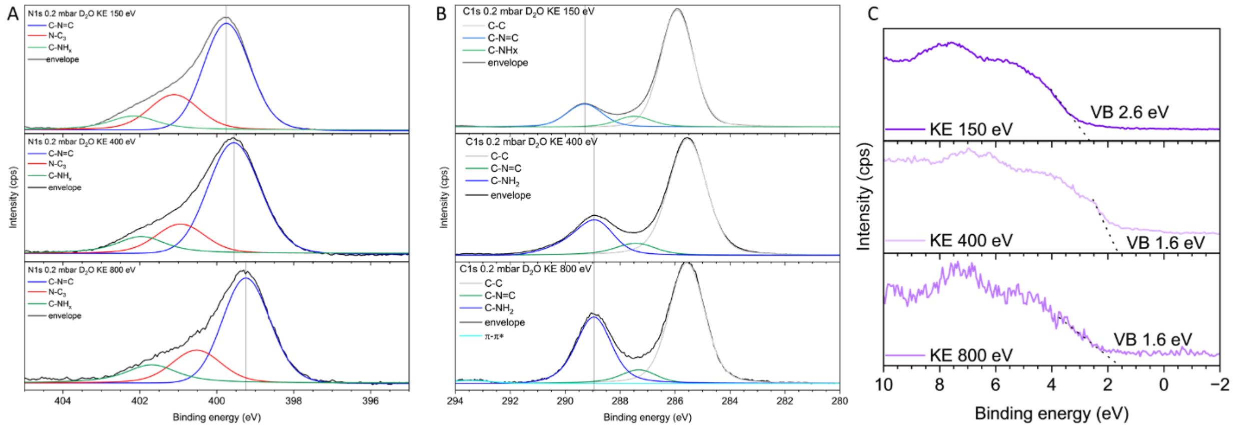

Figure S16. In-situ deep-profiling study of carbon nitride surface during water adsorption. XPS spectra of (A) N1s, (B) C1s, and (C) VB-XPS measured at kinetic energy 150 eV (top panel) 400 eV (middle panel) and 800 eV (bottom panel) under flow of 0.2 mbar D_2O.

Supplementary note 2:

Effect of different vapors

We studied the effect of the following solvents with different ε (methanol (ε=32.7), isopropanol (ε=19.9), and toluene (ε=2.4), in addition to D_2O (ε=78.1 at 25° C) at the pressure of 0.2 mbar on the electronic properties of carbon nitride. The recorded XPS spectra are shown in Figs. S17-S19.

Upon methanol adsorption (Figure S17 A-D), we observe a shift in the N1s features towards higher binding energies (0.5 eV for C-N=C, 0.4 eV for $N-C_3$, and 0.2 eV for $C-NH_x$), indicating electron density donation from nitrogen atoms to the methanol (Figure S17 A). This occurs due to hydrogen bond formation between the hydrogen atom of the hydroxy group (acceptor) in methanol and the nitrogen atoms (donors) of the carbon nitride. Consequently, the overall electron density of the semiconductor decreases, as reflected by the shift in the carbon spectroscopic features towards higher binding energies (Figure S17 B). Additionally, the arising of an O-H feature in the O1s spectrum is observed (Figure S17 C). The electron density donation into hydrogen bonds with methanol also causes a shift in the valence band position of the carbon nitride film by about 0.3 eV, lower than that of heavy water (Figure S17 D). This indicates that polar solvents like methanol can form a hybrid semiconductor structure with carbon nitride, resulting in significant electron density redistribution. We attribute the lower shifts recorded here to the lower dielectric constant of the methanol with respect to (heavy) water, inducing a weaker displacement of the electron density of the carbon nitride surface.

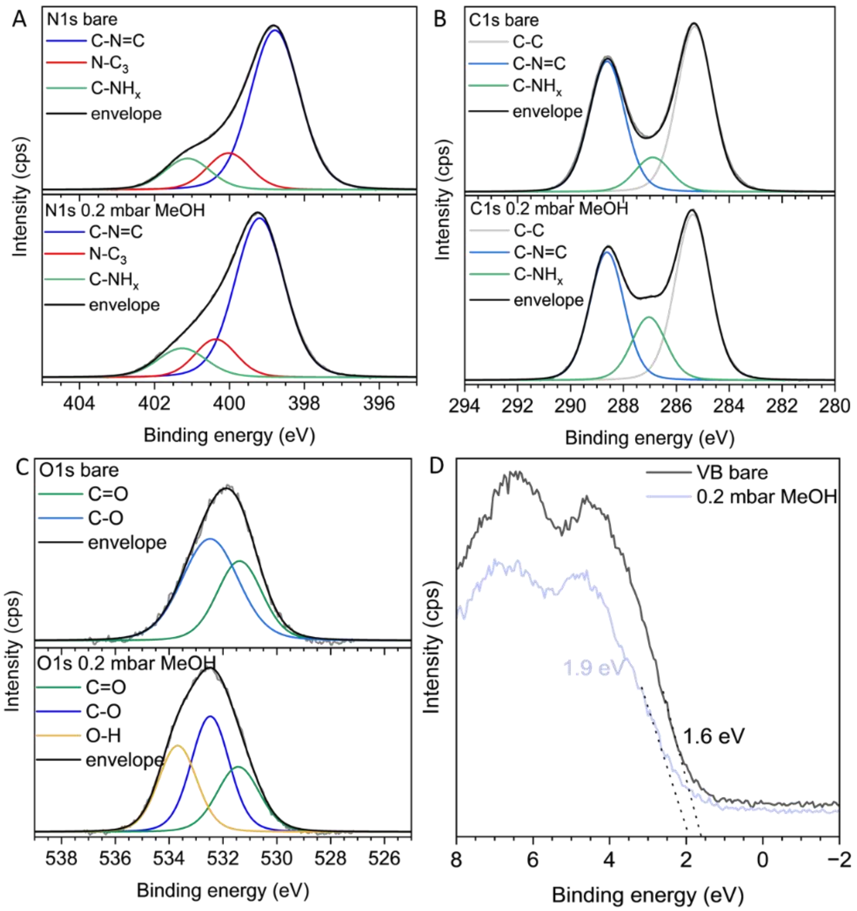

Figure S17. Methanol in-situ adsorption study. XPS spectra of (A) N1s, (B) C1s, (C) O1s and (D) VB-XPS of bare carbon nitride (top panel) and carbon nitride under flow of 0.2 mbar methanol (middle panel).

Isopropanol adsorption causes lower shifts in the C1s and N1s spectra (Figure S18 A, B). In the N1s spectrum (Figure S18 A), the N-C_3 feature shifted approximately 0.2 eV towards higher binding energy upon isopropanol adsorption, due to electron density donation from the carbon nitride's nitrogens that act hydrogen bond donor with the H(O)- of the isopropanol. The adsorption of isopropanol is also evident from a decrease in the relative intensity of C-N=C features with respect to the C-C one, in the C1s spectrum, due to the C-C bonds present in the adsorbed molecules. In the O1s spectrum, we record the arising of an additional feature from the introduction of additional oxygen-containing species, e.g. O-H groups, on the carbon nitride surface. Moreover, changes in the VB-XPS spectrum reveal a shift of approximately 0.3 eV towards higher binding energy after adsorption, attributed to the formation of hydrogen bonds and the consequent donation of electron density from carbon nitride to the isopropanol. Notably the shift is similar to that of methanol potentially attributed to the similar functionalities and relatively close ε values as compared to heavy water. These observations support the statement that the interaction of carbon nitride with solvents, especially those that can act as hydrogen bond donor, is not limited to water

and it influences its surface electronic properties. Furthermore, the XPS shifts induced by the solvent are in good agreement with the solvent dielectric constant.

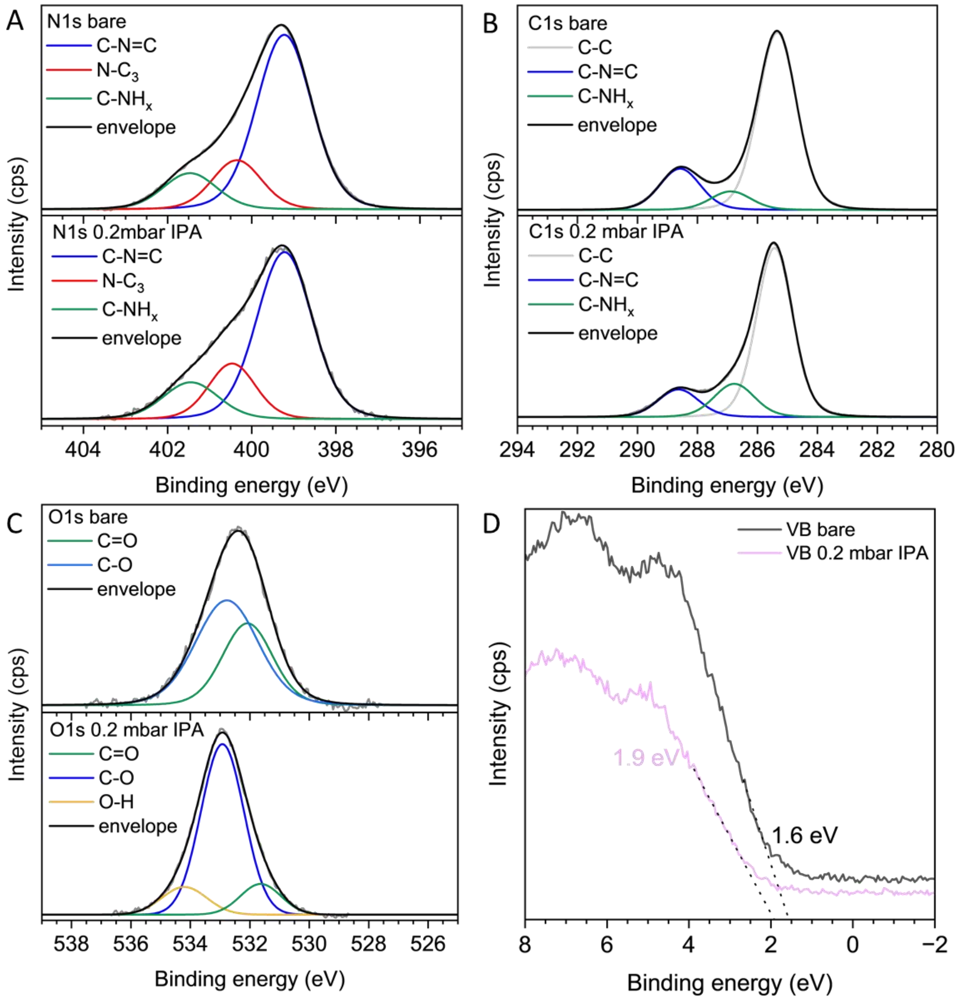

Figure S18. Isopropanol in-situ adsorption study. XPS spectra of (A) N1s, (B) C1s, (C) O1s and (D) VB-XPS of bare carbon nitride (top panel) and carbon nitride under flow of 0.2 mbar isopropanol (bottom panel).

The introduction of toluene vapors into the system (Figure S19), causes noticeable changes in the N1s spectra, where the carbon nitride features shifted by approximately 0.2 eV towards higher binding energies. This is likely due to weak van der Waals interactions and π-π stacking between the π-electron system of toluene and nitrogen atoms in the carbon nitride matrix. These interactions reduce the electron density around the nitrogen sites, leading to the observed binding energy shifts. A similar shift is observed in the VB-XPS spectrum of about 0.2 eV. The shifts in binding energies can also be attributed to the polarizability of toluene's π-electron cloud, causing electron density redistribution in the triazine ring. However, we do not record significant shifts in the C1s spectrum, besides a change in the C-C to C-N=C population as a result of the toluene surface adsorption

suggesting that the electron density at carbon atoms in the carbon nitride structure are less affected by the toluene for which the adsorption doesn't occur as a result of the formation of hydrogen bonds, but from weaker interactions such as π- π interactions.

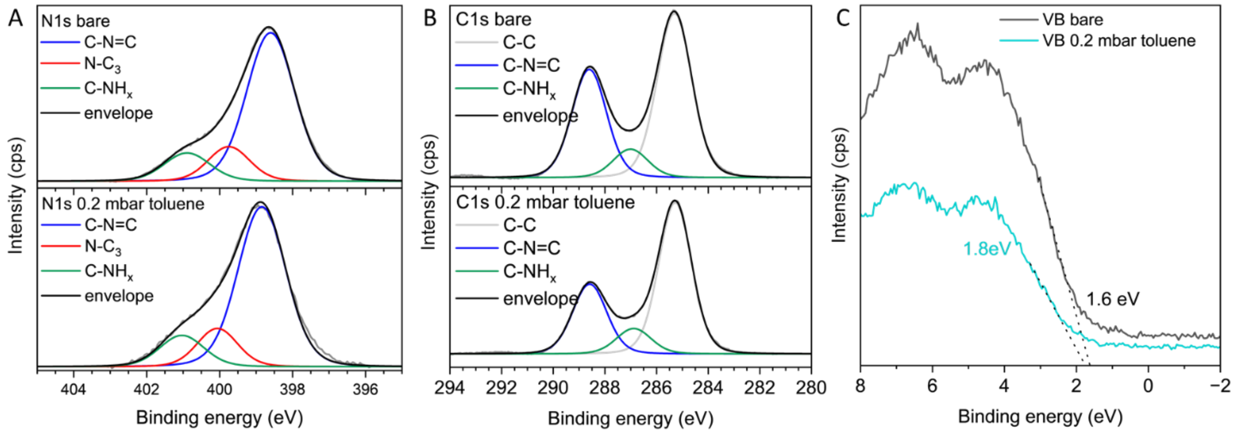

Figure S19. Toluene in-situ adsorption study. XPS spectra of (A) N1s, (B) C1s and (C) VB-XPS of bare carbon nitride (top panel) and carbon nitride under flow of 0.2 mbar toluene (bottom panel).

Eventually, solvents with higher dielectric constant and -OH groups can form hydrogen bonds with carbon nitride, leading to more pronounced changes on the surface electronic properties of the carbon nitride. On the other hand, in the case of toluene, a low dielectric constant solvent, a smaller change of the surface electronic properties has been also recorded, however, attributed to a different mechanism in the surface adsorption and attributed to weaker interactions, such as π- π interactions with the carbon nitride surface.

Supplementary note 3:

Stability of carbon nitride thin film over extended light illumination

Light stability is a critical factor in the performance of photocatalysts, as it determines their ability to maintain long-term photocatalytic activity under continuous light exposure. To assess this, we monitored the N1s, C1s, and VB spectral features of carbon nitride to evaluate the stability of its electronic structure during prolonged light illumination under photocatalytic conditions (Figure S20 A-C).

The N1s and C1s XPS spectra indicate that the overall structure of carbon nitride remains mostly unchanged after 2 hours of light illumination. This suggests that the carbon nitride framework is stable during photocatalysis, which is crucial for maintaining its long-term activity as a photocatalyst. However, a more detailed analysis of the C1s spectrum reveals a change in the population of the C-N=C feature with increasing illumination time up to 20%. We attribute this behavior to the desorption of surface contaminants, specifically adventitious carbon species, under prolonged illumination. The VB-XPS spectra do not show significant changes, supporting the hypothesis that the surface electronic structure of the carbon nitride remains stable during the photocatalytic process. This aligns with the stability observed in the core-level XPS spectra and the desorption of adventitious carbon species, which do not contribute to the electronic properties change on the carbon nitride film surface.

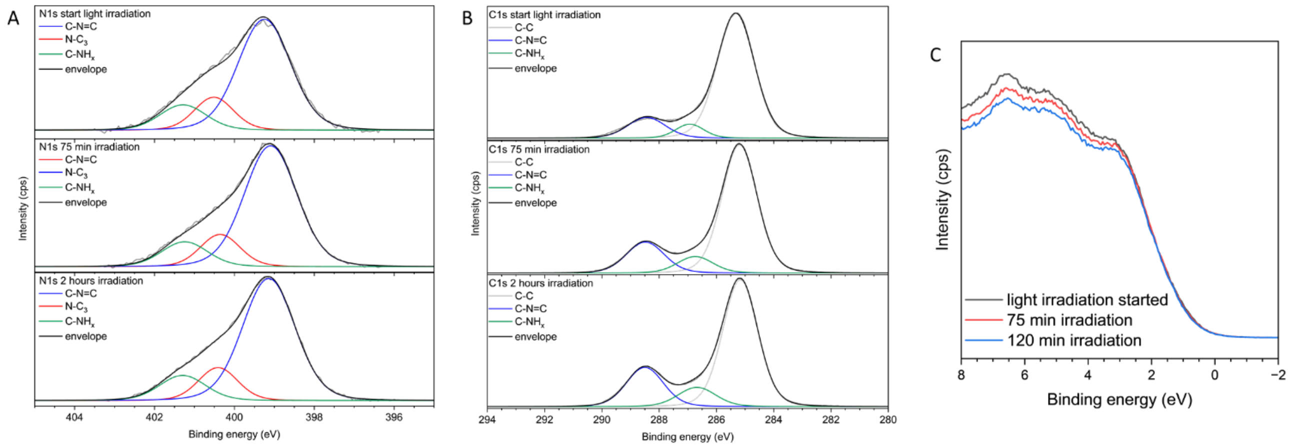

Figure S20. Study of electronic structure stability during continuous light illumination. XPS spectra of (A) N1s, and (B) C1s and (C) VB-XPS of carbon nitride under 0.2 mbar of D_2O at the beginning of light illumination (top panel), after 75 min of illumination (middle panel) and after 120 min of illumination (bottom panel).

Consequently, during the evaluation of the stability of the carbon nitride film for long illumination, we monitored the products formation using TOF-MS. Initially, we observed a gradual decrease in the signal associated with D_2O, accompanied by corresponding increases in the TOF-MS signals

for ^{-}OOD, D_2O_2, O_2, and D_2. After an initial stabilization, the system reaches a steady state, with a constant evolution of the products and negligible differences in the TOF-MS signals over the time of the experiment (about 2 hours). It is important to note that the signal for D_2O_2 was recorded and consistently present during the photocatalytic reaction, supporting the mechanistic hypothesis of a proton-coupled electron transfer mechanism (PCET), with the formation of D_2O_2 as an intermediate reaction product.

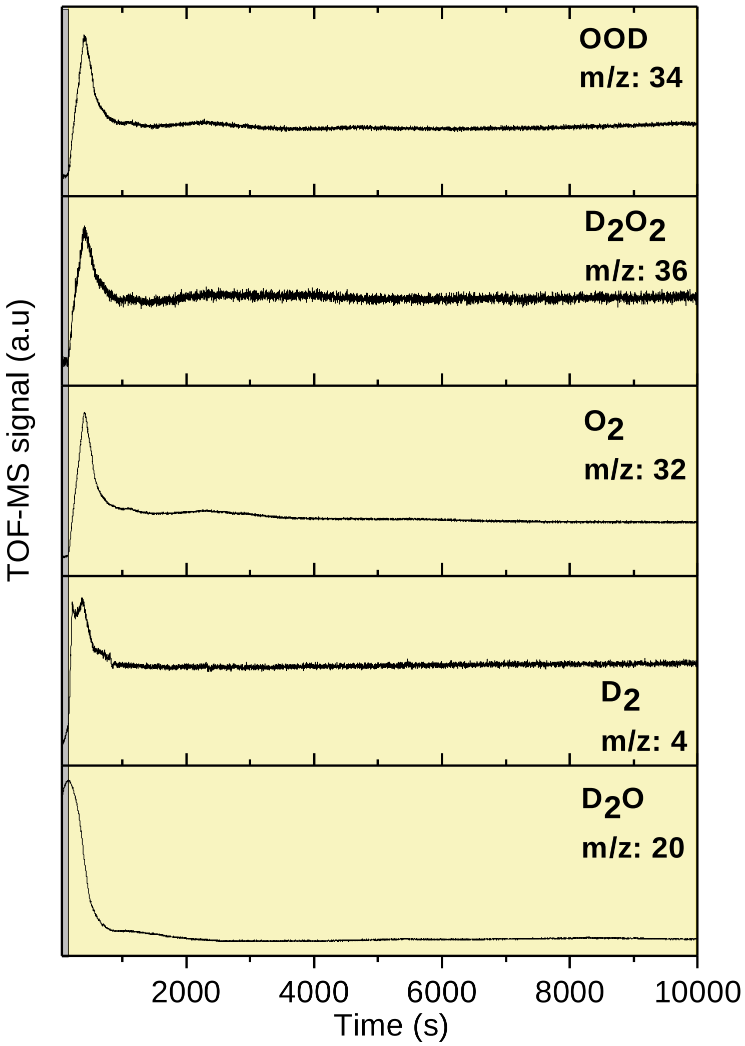

Figure S21. TOF-MS signals collected during photocatalytic study. From the top to the bottom: signal of ^{-}OOD, D_2O_2, O_2, D_2 and D_2O; grey areas indicate the absence of illumination; yellow areas indicate the time the carbon nitride film was subjected to illumination by means of the solar simulator.

Supplementary note 4:

<u>EPR spectroscopy</u>

To support proposed mechanism and confirm formation of radical, the radical species serving as reaction intermediates were analyzed by using an EPR spectrometer with 5,5-dimethyl-1-pyrroline N-oxide (DMPO) as a spin-trapping scavenger.

The presence of multiple peaks in the EPR spectrum under 415 nm light suggests the generation of radical species during the photocatalytic reaction (Figure S22, upper panel). The spectrum displays four main lines with a 1:2:2:1 intensity ratio, characteristic of a DMPO-OH adduct, indicating the presence of hydroxyl radicals (•OH), which result from trapping these radicals during the photocatalytic process (g factor 2.006). While the DMPO-OH adduct is prominent, there are also contributions from superoxide anions ($O_2{\bullet}^-$), trapped as a DMPO-OOH adduct (g factor 2.004), and from a DMPO-alkyl adduct (g factor 2.006). The absence of the sharp, narrow peaks typically associated with DMPO-alkyl adducts suggests that alkyl radicals may not be the dominant species, although their contribution cannot be entirely ruled out.[11]

The EPR spectrum recorded in the dark (Figure S22, lower panel) shows no significant peaks, confirming that the radical species observed under illumination are specifically generated by the photocatalytic activity of the carbon nitride thin film.

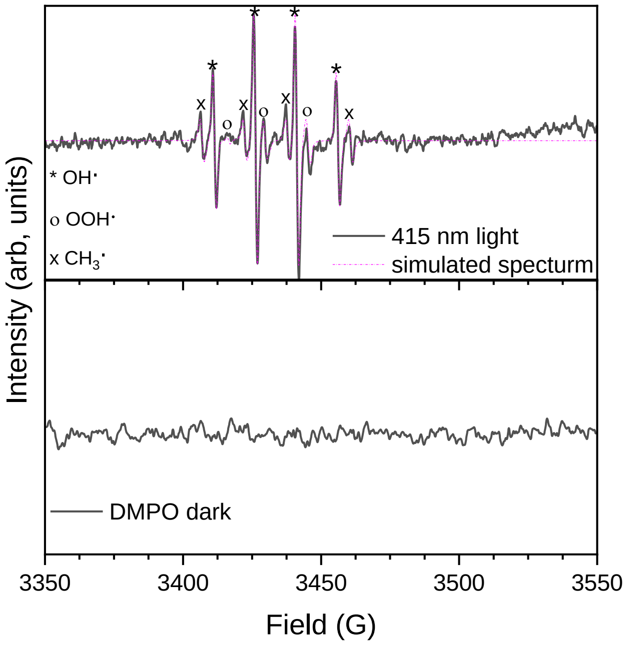

Figure S22. Radical trapping experiment using DMPO. EPR spectra of reaction mixture with DMPO during light illumination (upper panel) and in the dark (bottom panel).

Supplementary note 5:
<u>AFM characterization</u>

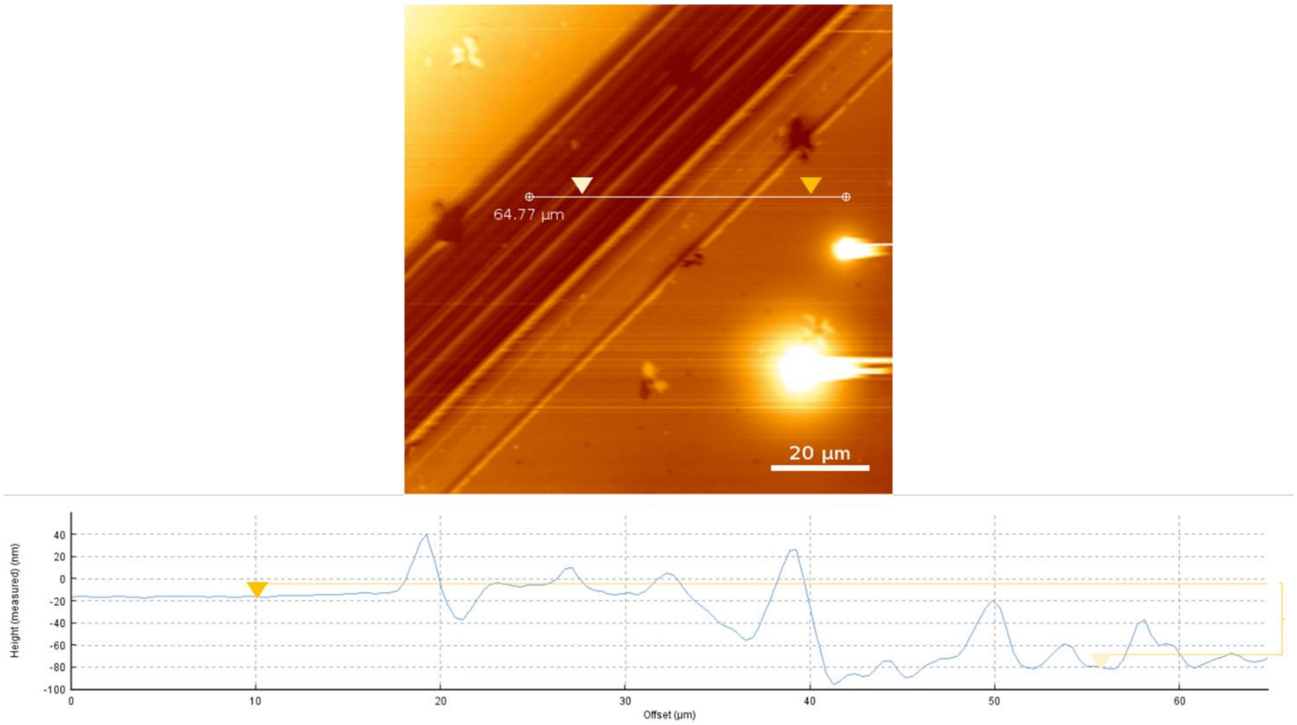

Figure S23. AFM step-height measurement. Bottom figure depicts the thickness of the prepared carbon nitride film of about 60 nm.

Supplementary References

1. Giusto, P. et al. Shine Bright Like a Diamond: New Light on an Old Polymeric Semiconductor. Adv. Mater. **32**, 1908140 (2020).
2. Akaike, K., Aoyama, K., Dekubo, S.. Onishi, A., Kanai, K., Characterizing Electronic Structure near the Energy Gap of Graphitic Carbon Nitride Based on Rational Interpretation of Chemical Analysis. Chem. Mater. **30**, 2341-2352 (2018).
3. Xie, D., et al. Key Role of Valence Band Position in Porous Carbon Nitride for Photocatalytic Water Splitting. J. Phys. Chem. C **126**, 14173-14179 (2022).
4. Qin, J., et al. Direct growth of uniform carbon nitride layers with extended optical absorption towards efficient water-splitting photoanodes. Nat. Comm. **11**, 4701 (2020).
5. Zhang, J.-R., Yet al. Accurate K-edge X-ray photoelectron and absorption spectra of g-C3N4 nanosheets by first-principles simulations and reinterpretations. Phys. Chem. Chem. Phys. **21**, 22819-22830 (2019).
6. Ren, J., et al. Role of Dopants on the Local Electronic Structure of Polymeric Carbon Nitride Photocatalysts. Small Methods **5**, 2000707 (2021).
7. Landoulsi, J., et al. Organic adlayer on inorganic materials: XPS analysis selectivity to cope with adventitious contamination. Appl. Surf. Sci. **383**, 71-83 (2016).
8. Smith, M., Scudiero, L., Espinal, J., McEwen, J.-S., Garcia-Perez, M., Improving the deconvolution and interpretation of XPS spectra from chars by ab initio calculations. Carbon **110**, 155-171 (2016).
9. Greczynski, G., Hultman, L., Impact of sample storage type on adventitious carbon and native oxide growth: X-ray photoelectron spectroscopy study. Vacuum **205**, 111463 (2022).
10. Lacombe, S., Bournel, F.. Laffon, C., Parent, P., Radical Photochemistry in Oxygen-Loaded Ices. Angew. Chem., Int. Ed. **45**, 4159-4163 (2006).
11. Wu, H.-Z., Liu, L.-M., Zhao, S.-J., The effect of water on the structural, electronic and photocatalytic properties of graphitic carbon nitride. Phys. Chem. Chem. Phys. **16**, 3299-3304 (2014).
12. Wirth, J., Neumann, R., Antonietti, M., Saalfrank, P., Adsorption and photocatalytic splitting of water on graphitic carbon nitride: a combined first principles and semiempirical study. Phys. Chem. Chem. Phys. **16**, 15917-15926 (2014)
